# De-risking clinical trial failure through mechanistic simulation

**DOI:** 10.1093/immadv/ltac017

**Published:** 2022-08-23

**Authors:** Liam V Brown, Jonathan Wagg, Rachel Darley, Andy van Hateren, Tim Elliott, Eamonn A Gaffney, Mark C Coles

**Affiliations:** Wolfson Centre for Mathematical Biology, Mathematical Institute, University of Oxford, Oxford, UK; Kennedy Institute of Rheumatology, University of Oxford, Oxford, UK; Pharmaceutical Sciences–Clinical Pharmacology, Roche Innovation Center Basel, Basel, Switzerland; Centre for Cancer Immunology, Institute for Life Sciences, University of Southampton, Southampton, UK; Centre for Cancer Immunology, Institute for Life Sciences, University of Southampton, Southampton, UK; Centre for Immuno-oncology, Nuffield Department of Medicine, University of Oxford, Oxford, UK; Wolfson Centre for Mathematical Biology, Mathematical Institute, University of Oxford, Oxford, UK; Kennedy Institute of Rheumatology, University of Oxford, Oxford, UK

**Keywords:** oncology, vaccines, mathematical modelling, peptide, late-phase trial

## Abstract

Drug development typically comprises a combination of pre-clinical experimentation, clinical trials, and statistical data-driven analyses. Therapeutic failure in late-stage clinical development costs the pharmaceutical industry billions of USD per year. Clinical trial simulation represents a key derisking strategy and combining them with mechanistic models allows one to test hypotheses for mechanisms of failure and to improve trial designs. This is illustrated with a T-cell activation model, used to simulate the clinical trials of IMA901, a short-peptide cancer vaccine. Simulation results were consistent with observed outcomes and predicted that responses are limited by peptide off-rates, peptide competition for dendritic cell (DC) binding, and DC migration times. These insights were used to hypothesise alternate trial designs predicted to improve efficacy outcomes. This framework illustrates how mechanistic models can complement clinical, experimental, and data-driven studies to understand, test, and improve trial designs, and how results may differ between humans and mice.

## Graphical Abstract

**Graphical Abstract:**
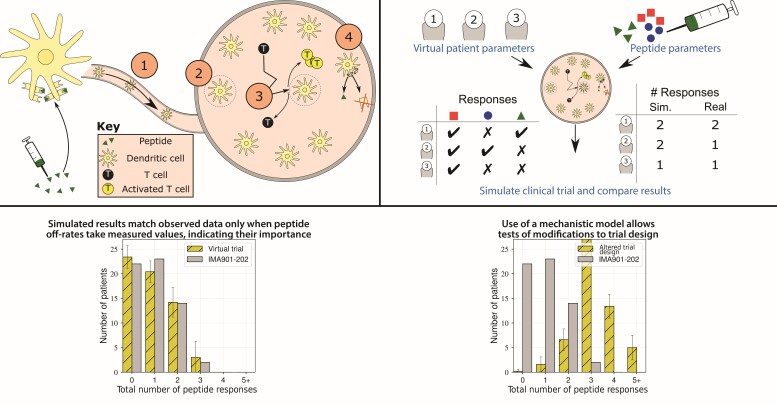

**Key points**

- IMA901 is a renal cell carcinoma cancer vaccine containing nine short peptides. No patient gave an immune response to more than three of the peptides.
- Simulations of IMA-901 were used to test the hypothesis that the response to short peptide vaccines could be limited by the off-rates of short peptides from major histocompatibility complex (MHC)-I.
- The hypothesis was found to be consistent with the results of IMA901.
- Results suggest that IMA901 could have been improved by reducing peptide competition or dendritic cell migration times, or alternative vaccine delivery strategies.
- Clinical trial simulations can identify important biological mechanisms and inform experiments that can improve the designs of clinical trials.
- Simulations based on mechanistic, rather than data-driven, models provide insight into mechanisms of action and can be used to generate and test biological hypotheses.

## Introduction

Many therapeutic agents fail in the later-phase II/III stages of development. With tens of thousands of clinical trials registered per year [1], a failure rate of 54% at phase III [2] and a cost of anywhere from $40m USD to $3b USD per trial [3], these failures cost the biopharmaceutical industry many billions of USD per year. It is therefore critical to understand whether early suboptimal or unexpected results are attributable to intervention design (such as dose scheduling and target patient population) and/or a therapeutic mechanism of action inappropriate for the targeted disease biology. *In silico* modelling is well-suited for this purpose, allowing quantitative and qualitative comparison of large numbers of patient or drug effects more quickly, cheaply, and ethically than *in vivo* study. It can narrow down the field of plausible hypotheses to guide experimental work and inform intervention design. There are two broad, extensively used methodologies that can be used to this end: data-driven techniques and mechanistic models. Data-driven techniques include machine-learning algorithms such as neural networks, which can be trained on data and used to predict the output of new inputs; such techniques are utilised by online shopping websites to provide customers suggestions based on previous purchases. An even simpler example is the calculation of the correlation of observables with different patient properties, which allows one to infer the importance of properties such as age, for example, by examining the correlation of outcome with age. Mechanistic models, on the other hand, are a set of equations or rules that directly describe relevant biology, with parameters that can be linked to observable quantities. The best known example in biochemistry is perhaps enzyme kinetics, which links the formation of enzyme–substrate complexes with reactant concentrations and affinity constants. Data-driven techniques can be used in a clinical context to predict how metrics such as overall survival may change with study design or in a different population (e.g. refs. [4, 5]). Despite their great predictive power, data-driven or statistical models do not generally contain a direct representation of the relevant biology and so are less well-suited to the study of hypothetical disease and drug mechanisms of action. Mechanistic models, which describe relevant biology with equations or simulations, are much better suited to *in silico* experimentation and hypothesis testing about mechanisms of action. Mechanistic model parameters usually have direct biological analogues, many of which can be controlled and tested in a clinical setting. One example of a mechanistic modelling study investigated the effect of bone morphogenetic protein treatment on paediatric disease of the bone [6], to understand the conditions under which disease severity is reduced by treatment and to stratify patients into responders, non-responders, and asymptomatic populations. An example applied to a clinical context predicted that short peptide cancer vaccines may preferentially select low-avidity T-cells, unless one optimises the dosage to reduce pMHC density on individual antigen-presenting dendritic cells (DCs) [7, 8]. However, compared to data-driven or statistical modelling, mechanistic modelling is less frequently used in a clinical context; a Web of Knowledge search for papers containing ‘machine learning clinical’ published between 2017 and 2021 yielded 17450 results, versus 4778 for ‘mechanistic model clinical’. The specific data-driven technique ‘neural network model clinical’ yields 7709 results. Nonetheless, mechanistic modelling is well-positioned to take advantage of the ever-increasing quantitative understanding of disease biology and mechanisms. In this study, we will demonstrate how *in silico* simulation of clinical trials can be used to test a biological hypothesis *in silico* to understand the mechanisms of clinical failure and improve upon trial designs, rather than merely to fit models to data. We will focus on published clinical trials of a short-peptide cancer vaccine, as an example.

It has been proposed that the immune system may not launch an attack on tumours that it has the potential to recognise because it lacks sufficient activating stimuli, due, for example, to inhibition of effector or antigen-presenting cells within the tumour microenvironment and its draining lymph nodes, or to a lack of stimulating peptide antigens. Invoking the cancer-immunity cycle of Chen and Mellman [9], the first three stages of the cycle are expected to contain a bottleneck to an immune response in this case. Therapeutic cancer vaccines contain tumour-associated peptides to ‘jump-start’ the cycle into a self-perpetuating anti-tumour response, have been a subject of study for many years [10], but have not yet seen wide clinical success. These vaccines encompass many mechanisms of action, from introduction of tumour-associated peptides to viruses engineered to express tumour antigen. Typical examples of the 1136 reported cancer vaccine clinical trials (as of July 2020) are the phase I–III clinical trials of IMA901 [11, 12], which is a renal cell carcinoma short peptide vaccine containing nine 9-amino acid peptides specific to HLA-A02* MHC-I. These peptides are tumour associated antigens, overexpressed on renal cell carcinoma cells. The vaccine showed clinical efficacy in phases I and II, as assessed by vaccine-induced immune responses against one or more peptides, though no patient responded to more than three of the nine peptides, and most responded to zero or one peptide(s). Furthermore, the trial failed in phase III when overall survival was used to assess efficacy, though the underlying reasons were unclear. It is feasible that a more robust immune response against a wider range of peptides may have improved this phase III outcome. However, it was uncertain as to which, if any, alternate clinical intervention design would have been capable of driving such improvements.

The purpose of this study is to test the hypothesis that the unbinding of vaccine peptides from antigen presenting cells due to differing pMHC affinities could limit responses to short peptide vaccines, by exploring whether a model based on this mechanism of action is consistent with observed patient data and with the generally greater efficacy of short peptide vaccines in mouse studies [13, 14]. We developed a series of *in silico* models of the vaccination site, the lymphatics and the lymph node, where the immune responses to short peptide vaccines are evoked. Like Kumbhari *et al.* [7, 8] and the phase I–II trials of IMA901, we use activation of an immune response as a surrogate for vaccine success. Unlike Kumbhari *et al.*, however, our aim is *not* to optimally fit trial data or to optimise model outputs. We aim to use our mechanistic model to run *in silico* clinical trials with the same designs as IMA901 phases I–III in order to test our hypothesis, by determining if our proposed mechanism of action is consistent with clinical observations. Similarly, we fit parameters that are expected to vary from patient to patient for each simulated patient in each phase of IMA-901 so as to match observed data, but we do not anticipate that the resulting patient parameter values would be the *only* values that could lead to a fit to data. Instead, we seek to determine whether the existence of a fit depends on peptide properties in a manner consistent with our hypothesis. A secondary aim of this study is to test potential changes to the designs of IMA-901 that may have yielded improved patient responses, in light of simulated results. This study demonstrates how *in silico*, mechanistic modelling can be used to propose and test biological hypotheses, a methodology that is invaluable as the amount and quality of quantitative biological and clinical data increases.

## Results

### Model summary: schematic of vaccination and T-cell activation

**Figure 1.**
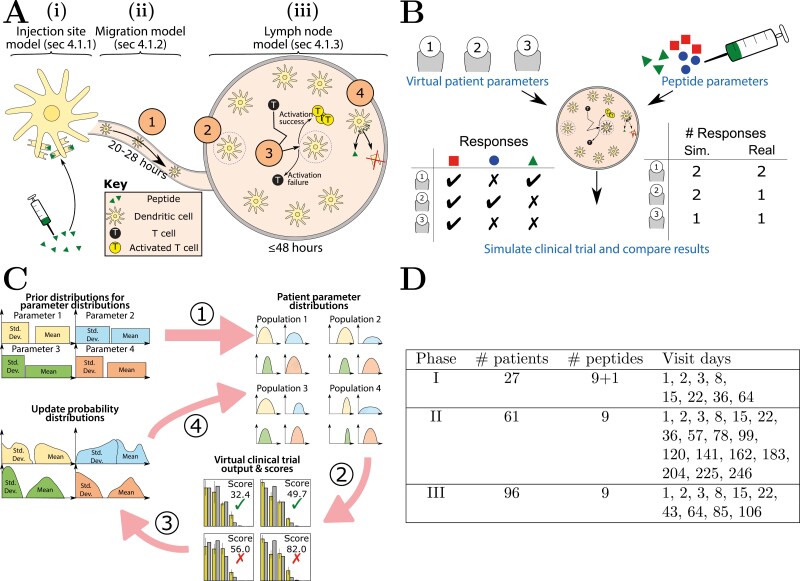
A summary of the model and methods used to conduct in-silico clinical trials, modified from [16]. (A) Model schematic, split into three sections. The injection site is modelled by a set of equations that represent peptide competition, binding to dendritic cell MHC-I and clearance from the vasculature. Unbinding of this initial amount of peptide from MHC-I is modelled in all sections of the model. Dendritic cell migration is modelled by a simple exponential distribution fitted to experimental measurements of their migration efficiency. T-cell–dendritic cell interactions in the lymph node are modelled by an agent-based model, see Methods: Computational model of vaccination. (B) Schematic of a simulated clinical trial. A patient cohort is produced with random values of patient-specific parameters. For each patient, the lymph node model is simulated with each peptide and the expected number of peptide responses is calculated. Three distinct simulated trials are performed to indicate the variability of results, and the numbers of patients responding to zero, one, two, or three peptides are compared to IMA901’s results. (C) Schematic of the data-fitting procedure using Approximate Bayesian Computation; see text in Results: Simulated clinical trial results for IMA901 phases I–I. (D) Quantitative details of each phase of IMA901: the number of patients enrolled, the number of peptides administered to each patient and the days on which peptides were administered (4 mg of each on each visit).

Induction of peptide-specific effector cytotoxic T lymphocytes by intradermal peptide vaccination requires peptide presentation by DCs to T-cells in the lymph node (LN) that drains the vaccination site. Peptides bind to DCs at the injection site and these cells then migrate to the LN to potentially drive an immune response against the peptides. Quantitative understanding of the relevant processes leading to immune response generation by effector T-cells requires an *in silico* model of the cellular and peptide interaction dynamics at the vaccination site (dermis), along the lymphatic vessels and within the draining LN. A schematic summary of the model is presented in Fig. 1A and described in detail in Methods: Computational model of vaccination. A complete list of parameters and assumptions are given in Tables 1 and 2. The model consists of three parts: the dermis, the lymphatics, and the lymph node.

**Table 1.** Complete list of model parameters

Sym	Parameter	Patient/peptide specific
*A*	**Fraction of MHC-I bound in dermis**	**Peptide**
*b*	Contact radius	Biophysical
*D*	**DCs migrated to lymph node**	**Patient**
*F*	T-cell free path	Biophysical
*F*_DC_	DC mean free path	Biophysical
*k*_off_	Peptide off-rate from MHC-I receptors	Peptide
*N*	MHC-I in T-cell–DC contact region	Biophysical
*P*	**Time of first DC arrival** (see caption)	**Patient**
*p*	**Spread of dermal departure times**	**Patient**
*ϕ*	**T-cell precursor frequency**	**Patient**
*ρ*	Density of T-cells in paracortex (see caption)	Patient
*r*_tot_	Number of MHC-I receptors per DC	Biophysical
*R*	LN (paracortex) radius	Biophysical
*T*	T-cell activation threshold	Biophysical
*v*	T-cell velocity	Biophysical
*w*	DC velocity	Biophysical

Peptide and patient-specific parameters that are varied to fit data are marked in boldface. Peptide off-rates from MHC-I *k*_off_ are fixed for specific peptides of interest, to measured/simulated values. The number of antigen-specific T-cells in the lymph node paracortex is equal to *ϕ* × *ρ*, and as only the overall number impacts model outputs, only the precursor frequency is varied. The time of the first dendritic cell arrival *P* is equal to the time that the first cell leaves the dermis plus the lymph transit time, hence only the latter is varied. The values and references for parameters that are not varied have been previously published [16].

**Table 2.** Key assumptions made in the model (see ref. [16] for more information.)

**Figure 2,:**
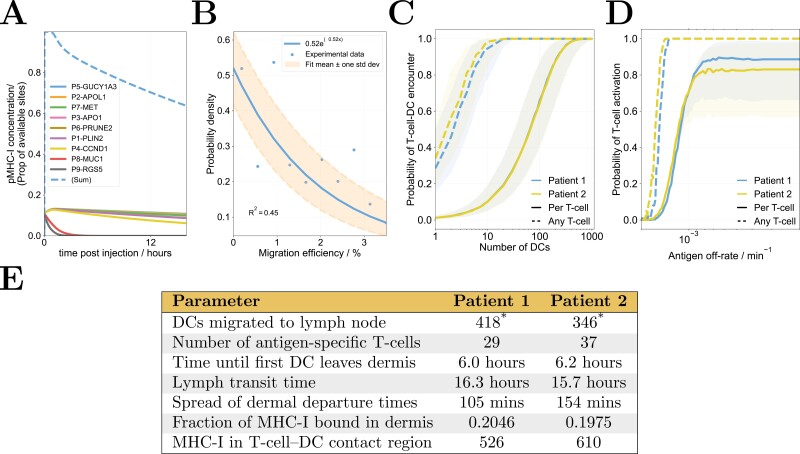
(A) Example results of the injection site model, showing peptide binding, the clearance of remaining free peptide from the vasculature and gradual unbinding of remaining peptides according to their off-rates. (B). Experimental measurements of dendritic cell migration efficiency with a simple exponential fit. The number of dendritic cells that successfully reach the lymph node in each virtual patient is drawn from this distribution. (C,D). Example results of the lymph node model for two virtual patients: the probability of simulated T-cell activation as a function of (C) the number of cognate-antigen carrying dendritic cells, when every interaction leads to activation or (D) pMHC-I off-rate. Solid lines indicate the probability for each (cognate) T-cell and dotted lines the probability of at least one (cognate) T-cell interacting. The shaded regions indicate probabilities between 6 and 24 hours, whilst the central lines indicate the probability after 12 hours. (E). Parameter values corresponding to the two virtual patients in panels C and D. *Note that in panel C, interaction probability is plotted against the number of migrating dendritic cells, so the fixed value in the table is not used. Also in panel C, the off-rate of peptide from MHC-I is fixed to 0.

**Vaccine Site**
Vaccine components other than the peptides are assumed to be non-limiting.
The concentration of peptides at the vaccination site is assumed to take the constant value of 200 µM.
The number of dendritic cells (DCs) per square millimetre of dermis is assumed to be 600 [18], and the initial dispersion area of injected vaccine solution is assumed to be 0.6 cm^2^.
The concentration of DCs at the vaccination site is assumed to be 2 × 10^−7^ µM. The number of DCs that successfully migrate to the lymph node is assumed to be between about 1 and 4 as fit by an exponential distribution (Figure 2B [17]).
Peptide on-rates are assumed to be equal.
Short peptides are assumed to bind directly to MHC-I receptors, which typically present ‘self’-peptides produced within the DC.
Each inactivated DC is assumed to initially have 10^5^ receptors, for a receptor density of 2 × 10^−2^ µM.
Peptide is assumed to be cleared overwhelmingly by the vasculature rather than the lymphatics and so is not encountered by DCs as they move through the lymphatic vessels.
Any new MHC-I receptors up-regulated during DC maturation are assumed to be independent of the initial population of MHC-I receptors that could be bound to the short peptide. Together with the assumption that peptide is cleared rapidly by the vasculature and rebinding can be ignored, this means only the exponential decay of the initial cognate antigen proportion needs to be considered, not any other self-peptides.
DCs are assumed to begin to migrate after several hours, leading to no free peptide being present in the lymphatic vessels for typical off-rates. It is hence assumed that peptide rebinding can be ignored.
**Lymph Node**
The lymph node is assumed to be non-inflamed and thus focus is on the probability of a first successful encounter between naïve T-cells and DCs.
T-cells in the model are present in the lymph node at the beginning of the simulation, but DCs carrying varying proportions of peptide antigen are assumed to arrive gradually from the vaccination site at physiological rates.
DCs are assumed to reach the lymph node at a constant rate λ=(D−1)/p, where *D* is the total number of DCs and the last DC arrives at time t=p. A model in which arrival times are random has also been tested.
The assumed lifetime of activated DCs within the lymph node is 48 hours [40–45]
T-cell and DC velocities and mean free paths are assumed to be similar between mice and humans.
DCs are generally assumed to be stationary in the lymph node. This assumption has been tested for validity.
**T Cells and Interactions**
Cognate peptide–MHC-I complexes, self peptide-MHC-I complexes and T-cell receptors (TCRs) are assumed to be expressed uniformly on the surface of DCs and T-cells.
When a T-cell interacts with a DC, it is assumed to be activated with a (binomial) probability that depends on the amount of cognate antigen presented by the DC. As only the number of cognate antigen and the total number on the DC are important, all other self-peptides can be ignored.
Sampling of pMHC-I by the T-cell is assumed to occur with replacement, allowing use of the binomial distribution instead of the hypergeometric distribution. The difference between the binomial and hypergeometric distributions is no more than 0.1 for any input cognate antigen ratio *A*, for the assumed number of MHC-I in the T-cell–DC contact region.
DCs and T-cells have multiple interactions over several hours [40, 46–48] and ‘integration’ of multiple such interactions leads to the final activation state of T-cells [40, 46, 47, 49], but it is assumed that a minimum amount of cognate antigen is required for any given interaction to contribute towards activation. Noting that antigen on the DC surface is monotonically decreasing in time, we have the corollary that if the first of such interactions fails, then all of them will fail. This and other assumptions (such as the lymph node not being inflamed) mean that the model’s output is the **maximum** probability of T-cell activation.
The variation of TCR affinities for different antigen is ignored; it is assumed that there is a precursor frequency *ϕ* of T-cells capable of recognising the antigen with approximately equal affinity. This is equivalent to choosing a cut-off affinity beyond which T-cell activation is successful, and is consistent with our calculation of a maximum probability of T-cell activation.
Immunological response is assumed to be an appropriate surrogate of clinical efficacy.

- *Dermal injection site* (Fig. 1Ai): Peptides compete for sites on DC MHC-I receptors. It is assumed that short peptides may only be presented on MHC-I by direct binding, as it is unlikely for short peptides that enter the cell to reach the endoplasmic reticulum without cleavage by cytoplasmic peptidases. Cleaved short peptides would be too short for loading onto MHC-I. Free peptide is cleared from dermis by the vasculature (due to their low molecular weight [15]), after which the remaining peptide-MHC-I complexes dissociate exponentially due to normal ligand-receptor kinetics.
- *Lymphatic transit* (Fig. 1Aii): After several hours in the dermis, DCs migrate to the draining lymph node, carrying varying amounts of bound peptide.
- *Lymph node model* (Fig. 1Aiii): In the draining LN are T-cells that are cognate to any peptides bound to DC MHC-I. Simulated T-cells and DCs are represented in an agent based model, i.e. with physical cells whose movement and interactions are tracked over time, as previously reported [16]. T-cells that come into contact with DCs have a chance of activation that depends on the amount of cognate antigen bound to the DC and the threshold number of T-cell receptors that must be ligated for activation. The output of the model is the maximum probability of successful T-cell activation for each peptide, defined as the probability that a given T-cell has at least one activating interaction with cognate peptide-carrying DCs.

To simulate the results of a clinical trial, a cohort of virtual patients with random parameters (drawn from plausible biological values, as informed by literature measurements) is defined, equal in size to one of the phases of IMA901 [11, 12]. For each virtual patient in turn, the maximum probability of T-cell activation over the course of the entire trial schedule is calculated. The number of patients and the schedule of each phase of IMA901 are displayed in Fig. 1D. We may calculate the number of patients expected to respond to 0, 1, 2, or 3 peptides from the simulated clinical trials and compare them to the results of IMA901. To test whether the model is consistent with those results, we used Approximate Bayesian Computation to iteratively update patient parameter distributions (such as time spent migrating in the lymphatics) until simulated trial results fit the data, as presented in Fig. 1C. The numbered circles in the figures correspond to different parts of the process:

1. Random patient parameter distribution means and standard deviations are initially uniformly drawn over a range. Each sample from these distributions is a set of Gaussian distributions that describes a population of patients.
2. For each sample, patient parameters are drawn from the population Gaussians and used to simulate a clinical trial. Each trial is assigned a score equal to the root mean square difference between simulated and observed results of IMA901. Low scores are accepted and high scores rejected.
3. The accepted parameter sets are used to change the probability distributions for the means and standard deviations of population parameter distributions.
4. The process is repeated until convergence with IMA901.

Our hypothesis was that the off-rate of vaccine peptides from MHC-I are the limiting factor for patient responses to each short peptide. We could test this by checking whether accurate replication of the results of IMA901 depends critically on the off-rates used for vaccine peptides, or not.

### Results: Summary of results from a study of the standalone model

A previous study of the standalone model [16] sought to investigate how the maximum probability of T-cell activation depended on model parameters. The assumptions that short peptides are cleared rapidly by the vasculature and that they must bind directly to MHC-I to be presented by DCs results in an initial population of peptide-MHC-I complexes that falls exponentially over time according to the peptide-MHC off-rate. For very fast off-rates, almost all peptide dissociates from MHC-I before the DCs reach the lymph node and the maximum probability of T-cell activation becomes zero. There is a sharp transition as this off-rate is lowered (or equivalently, the time taken for DC migration is lowered), to a region where further changes in off-rate have no impact, i.e. when it is low enough that there is sufficient peptide in the lymph node to activate T-cells for the entire lifespan of DCs in the lymph node. At such low off-rates, parameters such as the numbers of DCs and T-cells have an influence on T-cell activation probability. This behaviour can also be seen in Fig. 2D. This model suggested that peptide off-rates may be a key determinant of vaccine design, and led to this study, in which we test for consistency of this hypothesis with existing clinical data. A published sensitivity analysis of model outputs to various parameters [16] gives intuitive results: in the slow off-rate region, only factors relating to cell interactions (such as cell counts) are important. In the fast off-rate region, there is no T-cell response, and so nothing for which to measure the sensitivity of parameters. In the transition region, the model is sensitive to both cell and antigen-related parameters.

### Results: Predicted probability of vaccine response for an individual

We define a simulated patient by a set of random patient-specific parameters, for instance, the number of DCs recruited in the dermis. The immune response of each simulated patient is estimated from a model of T-cell activation, which considers the dermis, draining lymphatics, and lymph node, as detailed in Brown *et al*. [16]. Two example patients are considered in Fig. 2, with panel A showing the dynamics in the dermis. Here, multiple peptides compete with each other and endogenous (self) peptides for DC MHC-I sites, that are rapidly filled due to the large free peptide concentration. Free peptide is cleared by the vasculature from the dermis within hours and the distribution of peptides bound to MHC-I subsequently becomes determined by the peptide-MHC-I off-rate of each peptide. The presentation of most of the simulated peptides by MHC-I drops to a negligible level on a faster timescale than DC emigration from the dermis, and subsequently do not contribute to T-cell activation. In particular, note that all peptides form roughly similar proportions of initial pMHC-I complexes, including peptides 8 and 9 (MUC1 and RGS5), which have sufficiently short half-lives to ensure their lifetime in pMHC-I complexes is much shorter than the timescale of migration to the lymph node. Thus, the presence of such peptides is predicted in Fig. 2A to significantly reduce the antigen available for presentation.

Fig. 2B shows a probability distribution for the proportion of DCs that successfully migrate from the dermis to the lymph node, fit to clinical measurements [17]. These results show that fewer than 3% of DCs successfully migrate to the draining lymph node in any particular patient. Each simulated patient draws a random migration efficiency from this distribution. This random efficiency is multiplied by the DC density in the dermis (600 mm^-2^ [18]) and the assumed dispersion area of the vaccine (0.6 cm^2^) to define the number of DCs to be simulated in the lymph node for each vaccine dose and each virtual patient. Panels C and D show the predicted maximum probability of T-cell activation for two virtual patients, i.e. individuals with slightly differing biological parameters. Panel C shows the probability of activation against the number of successfully migrated DCs. Panel D shows it as a function of the off-rate of an antigen of interest. The typical number of DCs that migrate to the draining lymph node is predicted to be between 360 and 1440 (migration efficiency of 1–4%), and so response to a given peptide is predicted to be driven by its off-rate from MHC-I, not the number of migrating DCs. Results illustrate how the model is highly sensitive to antigen off-rates, with a ‘transition’ between a region where there is zero probability of activation and a region to where activation probability is finite but insensitive to further reductions in antigen off-rate. Peptides whose off-rates are far below this transition are those for which no response could ever be driven in the clinic and are not viable. Peptide off-rates in or near the transition region could drive a response in patients, depending on factors such as the migration time of DCs to the draining lymph node. Such factors could be controlled through clinical intervention design, e.g. by changing the mode of administration. In simulations of the clinical trial, peptides must compete for MHC-I sites on DCs, which increases the dependence of patient response probability on off-rates. A typical time course for the proportion of MHC-I molecules bound to peptides of interest is shown in Fig. 2A. Any off-rates significantly faster than other vaccine peptides, as for peptides 8 and 9 in the figure, will not evoke an immune response.

### Results: Simulated clinical trial results for IMA901 phases I–III

**Figure 3.**
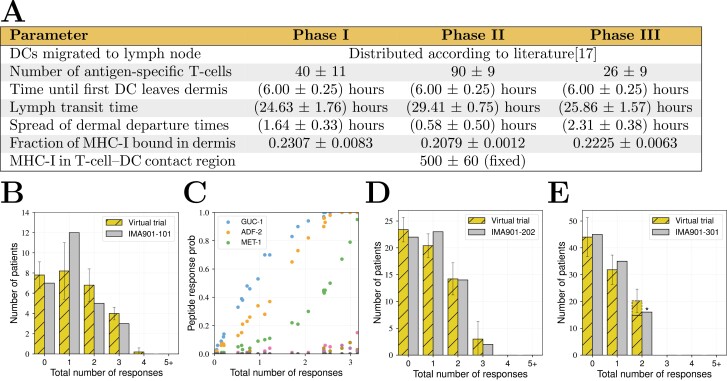
Results of fitting a simulated clinical trial to phases I–III of IMA901. (A) Parameters values for each patient in each phase are drawn from the Gaussian distributions indicated, the means and variances of which were fitted to data. The dendritic cell distribution is an exponential fit to literature data [17]. (B) The number of patients who responded to 0, 1, 2, or 3 peptides in the real and simulated trials of phase I. Error bars for the simulated trial are the standard deviation over three repeats. (C) The probability of a simulated patient responding to each peptide plotted against the total number of responses that the same virtual patient made in phase I. For example, a virtual patient expected to respond to 2.0 peptides has around a 90% chance of responding to GUC-001 and an 80% chance of responding to ADF-002. (D, E) Results of the simulated clinical trials that match phase II (D) and phase III (E). Note that the parameter distributions are different to each other and to phase I, and (*) that the number of patients responding to two or three peptides are combined in the data for IMA901-301 (phase III). The dashed bar is the prediction of the number of patients who respond to 3 peptides in the simulated trial.

To test the hypothesis that peptide off-rates drive the patient response distributions seen in IMA901 phases I-III, the model was used to simulate clinical trials with the same design as the phases of IMA901, as shown in Fig. 1B and as described in detail in Methods: Simulated clinical trials. We fit simulated results to observed data by varying the distributions of various parameters that are expected to vary from patient to patient, which are highlighted in bold-face in the list of parameters in Table 1. The resulting fits to the data are shown in Fig. 3. Panel A shows the mean and standard deviation of parameters for the patients in each phase of IMA901. These distributions differ slightly for each phase. The distributions for phases II and III are similar compared to the distribution for phase I (see Supplementary Appendix A). Panels B, D, and E show the number of patients that respond to 0, 1, 2, or 3 peptides in the real and simulated trials of IMA901; the simulated results are consistent with those of IMA901. Panel C shows the probability that a virtual patient responds to each specific peptide against the total number of peptide responses made by that virtual patient. The peptides GUC-1, ADF-2, and MET-1 are predicted to yield the most patient responses.

Notably, the fits in panels B, D, and E could only be achieved when peptide off-rates were assumed to take their measured values. The model could not be fit to data when values predicted by NetMHC or Bioinformatics and Molecular Analysis Section (BIMAS) [19, 20] were used for peptide off-rates, indicating that the hypothesis that the off-rates are critical for response is consistent.

### Results: Measurement of IMA901 peptide off-rates

**Figure 4.**
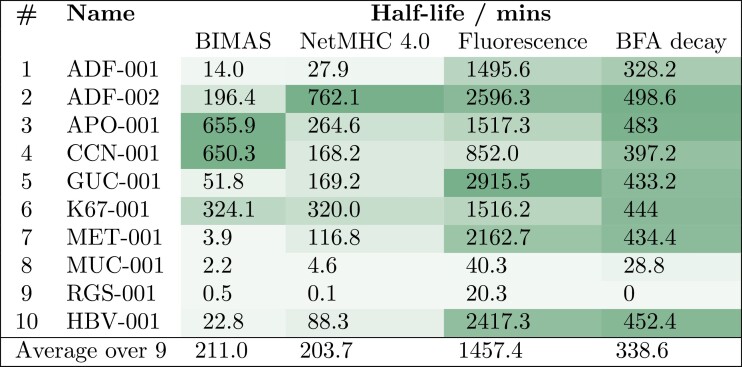
A comparison of the half-lives for each peptide, according to two algorithms ([19, 20]) and measurements by two techniques. The darker the cell shading, the higher the value in that column. ‘Fluorescence’ and ‘BFA’ refer to assays used to measure half-lives, as detailed in Results: Measurement of IMA901 peptide off-rates. Note that the rank order of each peptide differs for each technique, and that the average half-life measured by experiment differs greatly from the predicted values.

Simulated clinical trials could not be fit to the results of IMA901 phases I-III if estimates of the peptide off-rates from utilities such as BIMAS or NetMHC 4.0 were used (Supplementary Appendix C). Instead, the half-lives of the peptides of IMA901 were measured (Fig. 4). They were measured with a fluorescence polarisation assay repeated at two different concentrations of fluorescent peptide (FLPSDC*FPSV; see Methods: Measurement of peptide-MHC-I off-rates) and an MHC-I flow cytometry assay (BFA decay). The measured values are very different from those predicted by BIMAS and NetMHC 4.0, both in order of magnitude and in the distribution of peptide half-lives. However, the experimental values also differ from each other. The BFA decay data predicts a more uniform distribution of half-lives than the fluorescence experiment; five peptides have very similar half-lives, and the remaining two – judged to be very poor binders by every other measure – have a significantly lower or undetectable half-life. The fluorescence polarisation assay measured the binding of fluorescent peptide to immobilised MHC-I as bound peptides of interest unbind, which may be less indicative of *in vivo* behaviour than the BFA decay assay, which measures the rate of loss of MHC-I from the surface of B-cells. However, previous authors have shown that MHC-I recycling is faster in B-cells than in DCs, which are the cells of interest [21], and the BFA decay assay specifically measures the loss of pMHC-I complexes, not of peptide, and so may not be a good estimate of peptide off-rates from the MHC-I molecule. Furthermore, the fluorescence polarisation experiment had a much higher dynamic range (signal-to-noise ratio) than the B-cell BFA decay experiment or a repetition of the fluorescence polarisation experiment with 24nM of fluorescent peptide, rather than 12 nM. Hence, we only present the 12 nM fluorescence polarisation data in the fluorescence column of Fig. 4, and this is the data used for all experimentally based estimates of IMA901 peptide off-rates and presented results, unless otherwise stated.

### Results: Simulation of potential improvements to intervention design

**Figure 5.**
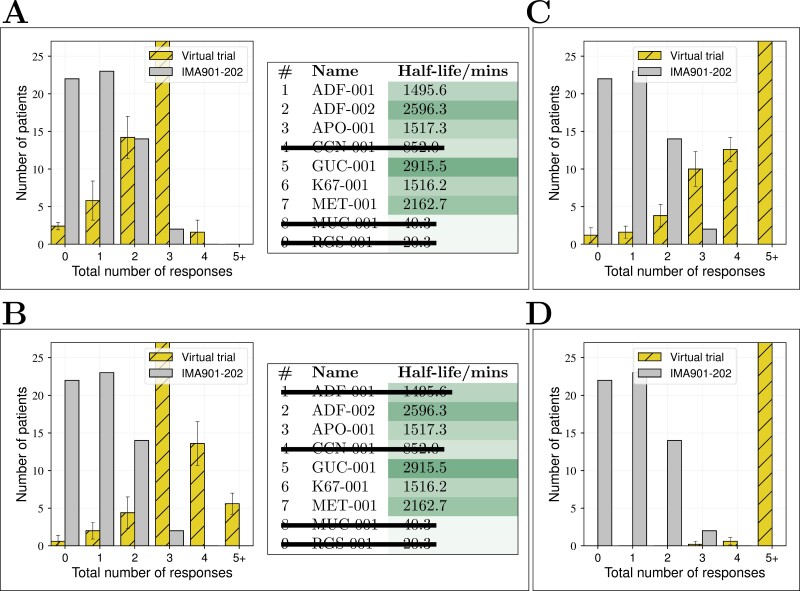
Tests of potential alterations to the intervention design to improve patient responses. Grey bars indicate the observed results of the phase II trial of IMA901. Simulated trial results match these results (as in Fig. 3D) with the parameters specified in Fig. 3A. The yellow bars in each panel indicate how results change with each of the following alterations: (A) the 3 peptides with the fastest measured off-rates are removed, (B) the 4 peptides with the fastest measured off-rates are removed, (C) lymph transit time is reduced from 30 to 10 hours (while also noting that transit times of alternative liposomal vaccine formulations from muscle to lymph are dramatically reduced), and (D) the changes in both B and C.

Previous work [16] and results presented so far indicate that simulated patient responses to short peptides are particularly sensitive to peptide–MHC-I off-rates, the number of available MHC-I sites for peptide presentation and the amount of time DCs spend migrating from the injection site to the draining lymph node. These parameters all affect the amount of peptide presented to T-cells in the lymph node and can be manipulated by clinical intervention design. We used our model to test the effect of various alterations to intervention design on virtual patient response probabilities, by comparing the results of a simulation of IMA901 phase II. Several of the measured half-lives are much lower than the others and are predicted to never evoke a response in virtual patients, as noted in the discussion of Fig. 2A above. We tested a vaccine from which these ineffective peptides were removed. In this case, competition for MHC-I sites during the loading phase is reduced and there is a greater initial number of sites available for the remaining peptides. This results in improved response distributions, as shown in Fig. 5A and B.

We also tested a scenario in which the migration time from the vaccination site to the draining lymph node is reduced to values appropriate for drainage from mouse dermis or, with further reductions in transit time, of an alternative formulation from human muscles, shown in Fig. 5C. This results in a much longer amount of time for T-cells and DCs to interact before peptide-MHC-I complexes dissociate, and so too results in an improved response distribution. Finally, we tested a design in which migration times and the number of peptides are reduced, in which case nearly all patients are predicted to respond to all five remaining peptides (Fig. 5D).

## Discussion

In this study, we have presented a framework with which one can test the consistency of a hypothesised mechanism of clinical failure with observations. We employed a mechanistic model to capture the dominant behaviour of the biological system, rather than using a more statistical approach to identify the correlates most associated with outcome. This allows identification of important biology without encoding large numbers of parameters and effects of unknown size. Our aim was to test the consistency of a proposed mechanism (that peptide off-rates from MHC-I are critical for short peptide vaccine success) with observed data, for which mechanistic models are better suited than more statistical approaches. In other words, it is whether *a* fit is possible that is important, not what ‘the’ fit is.

To illustrate this approach, we have simulated the short peptide vaccine clinical trials, IMA901 101, 202, and 301 [11, 12]. This required the development of a mechanistic model describing short peptide vaccination and T-cell activation from the dermis to the lymph node. The general behaviour of this model has been previously published [18], but results and discussion of assumptions are summarised in Methods: Computational model of vaccination for completeness. Each realisation of the model represents a virtual patient with unique parameters. Two virtual patients are compared in Results: Predicted probability of vaccine response for an individual. Model results are highly sensitive to peptide off-rates and related parameters (as presented previously [16]), for example, the initial amount of bound peptide and the time taken for DCs to migrate to the draining lymph node. This follows from the assumption that peptides bind directly to MHC-I and are not processed by the normal antigen presentation machinery, as any short peptide taken up by DCs would have to reach the endoplasmic reticulum without being cleaved by peptidases to do so. As supported by the results of Figs 2A and 5A, this leads to the hypothesis that the results of IMA901 were impacted by peptide competition in the dermis and the fast off-rates of several of the peptides. The probability of immune response calculated from the model is sensitive to peptide off-rates only when peptide half-lives are similar to or less than the timescale of DC migration to and persistence in the lymph node; small changes to very long half-lives will not impact the overall amount of peptide bound to MHC-I in the lymph node. Sensitivity to peptide off-rates is a necessary but not sufficient requirement for the model to support our hypothesis that the unbinding of vaccine peptides from antigen presenting cells due to differing pMHC affinities could limit responses to short peptide vaccines, as model sensitivity to off-rates does not guarantee that off-rates could explain clinical observations. Simulations of the clinical trial IMA901 over a cohort of virtual patients led to with model predictions that were consistent with observed outcomes. Moreover, simulated trial results are **only** consistent when peptide off-rates take their measured values rather than estimated values obtained from online utilities such as BIMAS and NetMHC 4.0 (see Supplementary Appendix C), which reinforces the hypothesis that short peptide off-rates are critical for vaccine response, and makes it less likely that the fit is trivial or a simple result of overfitting with numerous parameters. We note that the model could not have predicted the results of phase III using only the results of phases I and II, as it was not intended to be a predictive model. It could, however, have implicated peptide off-rates and competition as a potential limit on vaccine efficacy, in line with our study aims.

Off-rates predicted by online utilities were inconsistent not only with measured values but with each other, both in order of magnitude and in rank order of peptides, though they correctly predicted which peptides would bind poorly. Previous authors have made similar observations; a recent study evaluating performance of computational models in predicting CD8+ epitopes found that no existing algorithm performs ‘substantially’ better than random [22], and a study introducing MHCflurry, an MHC-I binding affinity prediction package that the authors present as an improvement over NetMHC and NetMHCpan, found that all three algorithms predict affinities several orders of magnitude away from measured values for most simulated peptides [23]. This shows that care must be taken when extrapolating the results of machine learning outside of the dataset used to train such models. As detailed in Results: Measurement of IMA901 peptide off-rates, we measured peptide off-rates by two different techniques and with two different concentrations of fluorescent labelled peptides, and expect that the measurements acquired from the 12 nM fluorescent assay is the most reliable, so use this for our estimates of IMA901 peptide off-rates, unless otherwise stated. Peptide on-rates were not measured with their off-rates. We have assumed that they are equal among vaccine peptides, noting that previous research has found that MHC-I affinity is determined mainly by the off-rate [24, 25], perhaps because the rate of the initial binding event is similar among peptides of equal size, or that it is diffusion-limited when peptide concentration is very large (as is the case in a vaccination).

We do not model T-cell receptor affinity for peptide-MHC molecules, instead assuming that there is a precursor frequency of all T-cells that could recognise each antigen. Defining a precursor frequency is equivalent to defining a proportion of T-cells that have at least a minimum affinity for a given peptide. This fits our strategy to seek the ‘maximum’ probability of T-cell response, rather than precise quantification of the extent of T-cell response. Low affinity T-cells can become activated after many interactions with antigen presenting cells, but we do not need to model this complexity if we instead quantify the number of T-cells whose affinity is just high enough to have at least one successful interaction. As we do not quantify the strength of T-cell responses, the use of T-cell receptor affinities instead of a ‘minimum affinity’ (precursor frequency) would not alter our conclusions. Similarly, though DCs present a variety of peptides, our use of the binomial distribution to calculate the probability that at least a minimum amount of antigen cognate to a given T-cell is present means that it is valid to ignore other peptides, including self peptides. See the list of assumptions in Methods: Computational model of vaccination for details.

Simulated clinical trial results were fit to those of IMA901 by altering input patient parameter distributions. Only a subset of model parameters was fit (see Methods: Computational model of vaccination); specifically, only those parameters that are controllable and expected to vary between patients were fit. That many patient parameters may be expected to vary among populations raises the question of whether the model has been overfit to data and whether a single ‘fit’ is meaningful. We aimed not to precisely predict the parameter values of IMA901’s patients (such as numbers of migrating DCs), but rather to test whether the proposed mechanism for short peptide vaccination failure (high off rates or peptide competition leading to a loss of pMHC-I complexes before T-cell activation) is consistent with observations. Indeed, there are many possible patient population distributions that could fit the data and care should be taken when interpreting parameter values of individual fits. For example, the number of antigen-specific T-cells was predicted to be lower in phase III than phases I-II (Fig. 3), but this is one fit of many potential fits and may not be a universal truth. Instead, comparisons should be made between the ‘posterior’ density plots of all possible parameters that lead to simulated trials that match observed results, as obtained from Approximate Bayesian Computation and shown in Supplementary Appendix A. In other words, model parameters are not identifiable, but this is not surprising, because activation of the immune system is a random process and must be robust to differing parameters values among different patients and different infection scenarios. Other authors have previously shown that biological model parameters are rarely identifiable and usually exhibit ‘sloppy’ sensitivities [26]. In such cases, one should instead focus on quantification of observable outputs and their uncertainty.

Comparison of the posterior distributions of possible patient parameters that could fit observed data (see Supplementary Appendix A) in each phase indicates that patient parameters distributions are equivalent between phases II and III of IMA-901, and so the observed difference in the number of peptide responses given by patients is predicted to be the result of randomness. However, the same analysis indicates that the patient population of phase I is distinct from the other two phases, at least in terms of parameter values. The prior anticipation was that the population in phase III would give the differing fit, as Sunitinib, which was given to all patients in the Phase III trial, has been (controversially) reported to reduce antigen presenting cell migration [27–29] and thus may reduce peptide response probabilities. There are multiple potential reasons for phase I’s required parameter values to be different. For instance, the difference may be due to the presence of a fluorescent peptide from Hepatitis B Virus in the vaccine in phase I. However, this difference would be expected to manifest as a requirement for more restrictive patient parameters to fit data, e.g. a smaller proportion of MHC-I bound to DCs leaving the dermis, and the predicted phase I parameter distributions are actually shifted in the opposite direction. Alternatively, it is possible that parameter distributions for phase I’s population are different because of the smaller sample size, which would mean that phase I’s results are an outlier and this would explain why simulated trial fits for phase I may appear to be worse than for phase II and III.

Use of a mechanistic model allows us to test alterations to IMA901’s intervention design, after fitting. Results in Results: Simulation of potential improvements to intervention design suggest that patient response distributions could be improved by reducing the number of peptides competing at the vaccination site, thus increasing the initial number of sites available to each peptide. We demonstrated this by removing peptides with the worst off-rates, although this was under the assumption that all peptides have the same on-rate to MHC-I. If these on-rates are different, the initial composition of peptides bound to MHC-I would differ and may thus alter the best choice of peptides to remove. The aim of IMA901 was to generate a broad anti-tumour response, hence the administration of multiple peptides. This requirement competes with that of reducing peptide competition for MHC-I sites. Though we predict that some peptides lead to stronger immune responses than others, especially due to variation in off-rates, it may be that less immunogenic peptides nonetheless yield a stronger anti-tumour response in particular patients. Furthermore, peptides with a higher T-cell precursor frequency may more readily induce T-cell activation than other peptides, but we have assumed that the precursor frequency of T-cells specific to each antigen is equal in each patient to avoid overfitting (see Methods: Computational model of vaccination). In both cases, it may not be appropriate to remove the peptides predicted to be the least immunogenic. Potential solutions for the competing aims of reducing peptide competition and encouraging a response to a broad repertoire of peptides are to target multiple human leukocyte antigen (HLA) sub-types, to use multiple vaccine sites (possibly draining to the same lymph node) and to increase the persistence of peptide in the dermis and/or within DCs. We also demonstrated improved predicted outcomes on reducing the amount of time that DCs spend migrating to the lymph node. Noting that transit times are reduced in mouse relative to human and are much lower in alternative liposomal vaccine formulations delivered to muscle, this suggests that efficacy in mouse models may be exaggerated and alternative delivery routes using different formulations may be beneficial. Furthermore, the prediction that the immune response to a vaccine is dependent on timescale and peptide competition is important as it suggests that short peptide vaccines are uniquely susceptible to the unbinding – and hence the fast off-rates – of vaccine peptides.

These results exemplify the ability of *in silico* studies to complement experimental and clinical investigation. Possible differences between mouse models and human patients can be identified and accounted for, alterations to intervention design can be tested before a final design is taken forward, and any model failures or missing biology can be easily identified and remedied. Mechanistic models are particularly well suited to identify potentially important biological mechanisms, which may be further investigated by experiment. The use of *in silico* modelling to identify problems before moving into larger trials has enormous potential to save resources and to reduce, replace, and refine animal experiments. *In silico* modelling is well established within the life sciences and in clinical studies (e.g. refs. [4–6]), but *in silico* experimentation and combined experimental-modelling studies are less common [30]. This approach holds great potential to accelerate discovery in human medicine in the coming years.

## Methods

### Methods: Computational model of vaccination

We developed an *in silico* model of a short peptide vaccination from the injection site (dermis) to CD8^+^ T-cell activation in the draining lymph node. A schematic of the model is shown in Fig. 1A, a list of its parameters and assumptions are given in Tables 1 and 2. Not all of the listed parameters are varied when fitting to data in the main text; only those that are controllable or are patient-specific. The model can be split into three parts: peptide–DC dynamics in the injection site, migration of DCs to the lymph node, and DC–T-cell dynamics in the lymph node [16].

### Methods: Peptide–dendritic cell dynamics in the injection site

The vaccine includes both peptides and adjuvant, which are both subjected to diffusion and clearance through the vasculature and lymphatics. Larger molecules diffuse more slowly, so the diffusion area of the adjuvant is more limited than the peptides. However, smaller molecules are cleared more quickly, primarily by the vasculature [15, 31–33]. The limiting factor for binding to DC MHC-I is hence the peptide rather than the adjuvant, so the latter is ignored in further modelling and DCs within the effective dispersal area of short peptides are assumed to be activated and migratory.

Peptide binding in the dermis is modelled by a set of ODEs,


$$\begin{array}{l} \begin{array}{ll} & \frac{dl}{dt}=k_{of\;f}c-k_{on}rl-k_{clear}l,\\ & \frac{dr}{dt}=k_{of\;f}c-k_{on}rl,\\ & \frac{dc}{dt}=k_{on}rl-k_{of\;f}c, \end{array} \end{array}$$ (1)

where *l*, *r,* and *c* are the concentrations of free peptide (**l**igand), free MHC-I **r**eceptors and bound peptide-MHC-I **c**omplexes, respectively, and *r* + c = *r*_tot_ is constant. These equations follow from the law of mass action, which has been extensively validated in many models, with a long history and a statistical mechanical basis [34]. They are particularly similar to the equations which are subsequently simplified (using low enzyme concentration and fast complex formation) to give Michaelis-Menten kinetics for enzymes, which share similar features to MHC-I binding.

These equations simplify in the context of a short peptide vaccination. Small molecules are cleared primarily through the vasculature, and for molecules of the size of nine amino acids, almost none are cleared through the lymphatics [15, 31–33]. In particular, clearance through the vasculature occurs on a timescale of minutes [31–33]) and DCs only begin to migrate after several hours, motivating our assumption that there is no free peptide present in the lymphatic vessels and that rebinding can be ignored, in which case these equations are effectively $\frac{dc}{dt}=-k_{of\;f}c$ in the lymphatic vessels. Perturbation analysis can also be used to show that this effective single equation also holds in the dermis after an initial transient timescale, in a similar manner to the reduction of law of mass action enzyme dynamics to Michaelis-Menten kinetics [35]. Note that to model nine peptides at once, *l*, *c,* and *k*_off_ can be replaced by peptide-specific variables such as *l*_*i*_ and *c*_*i*_, with 0 ≤ *i* < 9. In this case, the equations would be coupled because the total number of free and bound receptors is a constant, $r+\sum_{i=0}^{8}c_{i}=r_{tot}.$

### Methods: Migration of dendritic cells to the lymph node

Dendritic cells begin to migrate from the dermis to the draining lymph node after several hours. Experimental data [17] indicate that the migration efficiency (proportion of migrating DCs that are later found in the draining lymph node) is of order 1%. We fit an exponential distribution to the experimental data and draw random numbers of DCs from the fit for use in the lymph node model. The arrival time of DCs to the lymph node can be assumed to be random or linear, but previous work [16] indicates that this has little impact on results, so we assume that DCs arrive at a constant rate between the arrival of the first and last cells. The proportion of dendritic MHC-I that are bound to short peptides, *A*, is assumed to fall exponentially over time due to the peptide–MHC-I off-rate *k*_off_,


$$\begin{array}{l} \frac{dA}{dt}\left(t\right)=-k_{of\;f}A\left(t\right). \end{array}$$ (2)

### Methods: Dendritic cell–T-cell dynamics in the lymph node

The maximum probability of T-cell activation is calculated by a model whose details have been previously published [16] and which is shown in Figs 1A (schematic) and 2 (results). It is an agent-based model in which a defined number of simulated DCs are assumed to arrive at staggered times to the lymph node with a proportion of their MHC-I bound to a peptide of interest. This proportion falls over time as peptide–MHC-I (pMHC-I) unbind due to receptor binding kinetics (Equation 2). Once the DCs reach the paracortex of the lymph node, which is modelled as a sphere, they are considered stationary in comparison to T-cells. T-cells take realistic velocity and free path distributions (run-and-tumble or Levy walks, see ref. [16]), searching randomly within the lymph node until they make contact with DCs. Upon contact, the T-cells have a chance of activation: assuming that a minimum number of T-cell receptors must be ligated for successful interaction, the activation probability on contact is the probability that at least this number of pMHC-I are in the T-cell–DC contact area. The output of the model is the probability that at least one simulated T cell is activated. We make the following biological assumptions:

1. The probability of T-cell activation depends only on this first interaction ‘succeeding’; we ignore downstream events and assume that if this first interaction fails, then all subsequent interactions will also fail. We hence find the maximum probability of T-cell activation.
2. We ignore the varying affinity of T-cell receptors for different peptides. Instead, the model invokes the concept of a ‘precursor frequency’ of T-cells that can recognise a given peptide, to define the number of T-cells that should be simulated within the lymph node. This frequency is assumed to vary between patients.
3. The precursor frequency of T-cells is not assumed to be different for each peptide, in order to reduce the number of patient parameters to be fit. Had nine different variables been defined for precursor frequencies (for each peptide in IMA901), then it would be possible to manipulate values to fit any patient response distribution one wished, and the model could not have produced useful information.
4. We assume that short peptides are not transported onto MHC-I after intracellular uptake, as this process would require that they not be cleaved by intracellular peptidases. This assumption leads to direct binding as the only route for pMHC-I complex formation and leads to a strong dependence of results on pMHC-I off-rates and related patient parameters.

The values of many parameters, such as T-cell velocities, are defined using experimental literature. Other parameters, such as the number of DCs that migrate to the lymph node, may be expected to vary between patients. The strategy for handling these parameters is given in Methods: Simulated clinical trials.

### Methods: Simulated clinical trials

In order to determine whether model hypotheses are consistent with the results of IMA901, we ran simulated clinical trials using the lymph node model described in Methods: Computational model of vaccination. The procedure is shown in Fig. 1B and is as follows: a virtual patient cohort equal in size to one of the phases of IMA901 is created, and each patient is given normally distributed random values for each parameter that is expected to differ between individuals (T-cell precursor frequency, lymph transit time and spread, and the proportion of MHC-I available to vaccine peptide in the dermis) and that the underlying model is predicted to be sensitive to [16]. Dendritic cell migration efficiency for each patient is drawn from an exponential distribution fit to experimental data [17]. The system of ODEs described in Methods: Peptide–DC dynamics in the injection site is used to determine the initial abundance of each peptide, given values for the pMHC-I off-rates and assuming equal initial concentrations and on-rates.

The lymph node model is then run for each virtual patient and peptide in turn (using predicted initial abundances of each peptide), yielding a predicted probability that patients respond to each peptide after the entire vaccination schedule. The sum of these gives the expected number of peptide responses for each patient and in turn the number of patients expected to respond to 0, 1, 2, or 3 peptides, which can be compared to the data reported by the authors of IMA901. The means and standard deviations of the Gaussian distributions for patient parameters (e.g. there is a Gaussian distribution for possible lymph transit times, from which each patient draws a value) can be controlled to find a set of parameter distributions for which simulated trial results match the results of IMA901. For presented results, the lymph node model is repeated 10 times to reduce uncertainty of the probability of T-cell response. The entire simulated trial is repeated three times, each with different random patients, in order to gain an estimate of the variability of results due to stochasticity in patient parameters. We were not aiming to refine the mean output, hence more than three repeats were not required.

Although the phase I trial of IMA901 contains the marker peptide from HBV, the number of responses made to it are not reported or of interest. We wish to match the data on the number of responses made to the nine vaccine peptides to predicted responses, and hence HBV is not modelled – its presence would represent only a reduction in the maximum number of MHC-I sites bound in the dermis, which in any case is determined by fitting of parameters to the reported data.

### Methods: Measurement of peptide-MHC-I off-rates

#### Methods: Fluorescence of immobilised MHC-I

##### Production of MHC-I proteins.

A pHN1+ plasmid encoding the mature human beta 2-microglobulin protein (β2m hereafter) was obtained from Prof P Moss. A pET22b plasmid encoding HLA A*02:01fos was obtained as described in [36]. Peptide-loaded MHC-I complexes were obtained as in [37] by refolding solubilised inclusion bodies of MHC-I heavy chains with solubilised inclusion bodies of human β2m and UV-labile MHC class I specific peptide.

##### Peptides:

The following HLA-A*02:01 binding peptides were used: the UV-labile peptide KILGFVFjV (j represents 3-amino-3-(2-nitro) phenyl-propionic acid), the fluorescent peptide FLPSDC*FPSV (C* denotes TAMRA-labelled cysteine), NLV (NLVPMVATV), NAV (NAVPMVATV), ADF-1 (SVASTITGV), ADF-2 (VMAGDIYSV), APO-1 (ALADGVQKV), CCN-1 (LLGATCMFV), GUC-1 (SVFAGVVGV), K67-1 (ALFDGDPHL), MET-1 (YVDPVITSI), MUC-1 (STAPPVHNV), HBV-1 (FLPSDFFPSV), and RGS-1 (LAALPHSCL) were synthesised by GL Biochem.

##### Fluorescence polarisation experiments:

Fluorescent polarisation measurements were taken using an I3x (Molecular Devices) with rhodamine detection cartridge. Binding of TAMRA-labelled peptides is reported in millipolarisation units (mP) and is obtained from the equation $1000\frac{S-GP}{S+GP},$ where S and P are background-subtracted fluorescence count rates (*S* is the polarisation emission filter is parallel to the excitation filter; *P* is the polarisation emission filter is perpendicular to the excitation filter; and *G* (grating) is an instrument and assay-dependent factor). All experiments were conducted at 25°C in duplicate and used phosphate-buffered saline (PBS) supplemented with 0.5 mg/ml bovine gamma-globulin (Sigma), in a volume of 60 μl.

Peptide-receptive HLA-A*02:01fos complexes were obtained by mixing 75 nM HLA-A*0201fos loaded with UV labile peptides with 1.5 μM human β2m (Fitzgerald) and exposing to ≈360 nm light for 20 minutes at 4°C (‘UV exposed’). UV-exposed MHC class I molecules were incubated with 75 nM of unlabelled peptide overnight at 25°C. The next day 12 nM or 24 nM of FLPSDC*FPSV TAMRA labelled peptide was added and binding of FLPSDC*FPSV was measured for approximately 200 hours. Non-linear regression was performed, using the one phase association model in Prism.

#### Methods: B-cell MHC-I BFA decay

##### Cell lines.

The T2 cell line [38] was maintained in Tetramethyl Rhodamine (Sigma-Aldrich) with 10% foetal bovine serum (Globepharm), 2 mM L-glutamine (Sigma-Aldrich), and 10 mM N-2-hydroxyethylpiperazine-N’-2-ethanesulfonic acid (Lonza) at 37°C with 5% CO_2_.

##### Peptides:

ADF-1 (SVASTITGV), ADF-2 (VMAGDIYSV), APO-1 (ALADGVQKV), CCN-1 (LLGATCMFV), GUC-1 (SVFAGVVGV), K67-1 (ALFDGDPHL), MET-1 (YVDPVITSI), MUC-1 (STAPPVHNV), HBV-1 (FLPSDFFPSV), and RGS-1 (LAALPHSCL) were synthesised by GL Biochem and re-constituted in 100% Dimethyl Sulfoxide (DMSO) to give 10 mM stocks.

##### BFA Decay:

T2 cells were incubated in 24-well plates at 3 × 10^5^ cells/ml in AIM V Serum Free Medium (ThermoFisher) for 18 hours at 26°C with 5% CO_2_ + peptide at 20 μM or DMSO control. Peptide-loaded cells were washed with PBS to remove excess peptide and re-suspended in 0.2 ml AIM V Serum Free Medium containing Brefeldin A (Sigma-Aldrich) at 5 g/ml. Incubated in 96-well plates at 37°C with 5% CO_2_ for the specified time points. At the end of the time course each well was washed with PBS + 0.5% foetal bovine serum (Globepharm), stained with anti-HLA-A2 antibody BB7.2 [39] followed by goat anti-mouse conjugated with fluorescein isothiocyanate (Sigma-Aldrich) and analysed by fluorescence-activated cell sorting (BD Caliber).

##### Analysis:

Mean fluorescence intensity measurements were determined using FCS Express software. HLA-A2 was expressed as the percentage of mean channel fluorescence at time point 0. Half-lives were determined from non-linear regression curve fits using GraphPad Prism.

## Supplementary material

Supplementary data are available at *Immunotherapy Advances* online.

**Figure S1:** Two-dimensional projections (density plots) of the posterior distribution obtained through Approximate Bayesian Computation (see text). 10000 sets of random values for all parameters are taken from a ‘basket’ (prior distribution) of possible sets. Those that yield simulated clinical trial results closest to the real-world results are used to form a new basket. This process is repeated 25 times, yielding the posterior distribution shown. Areas of higher density indicate regions of parameter space that match the real-world data best. The density plots of phases I, II and III are shown together, allowing the difference between the phases to be visualised.

**Figure S2:** Two-dimensional projections (density plots) of the posterior distributions obtained through Approximate Bayesian Computation, where only two parameters are allowed to change at a time (indicated in each subpanel). This is unlike Figure S1, where every parameter is allowed to change at once and 2D projections of the resulting posterior distributions are plotted. As before, areas of higher density indicate regions of parameter space that match the real-world data best. Densities for phases I, II and III are plotted together in each panel. Note that the density of parameters that match phase I is distinct from those for phase II and III.

**Figure S3:** Results obtained by matching a simulated clinical trial to the phase I trial of IMA901, as in Fig. 3, but values used for the pMHC-I off-rates for the peptides in IMA901 are obtained from online utilities for estimating pMHC-I off-rates, BIMAS [20] or NetMHC [19]. The left plot of each pair shows one of the best fits (by the sum of squared differences between bar heights) for that algorithm. The right plot shows the result of greatly increasing the proportion of MHC-I bound by peptide in the dermis, in order to try and gain probability content in the next column along without losing probability content in the bar for 0 responses.

ltac017_suppl_Supplementary_Appendices

## Data Availability

The data underlying this article are available in the Oxford Research Archive, at https://dx.doi.org/10.5287/bodleian:YQQj4Kd87.

## Abbreviations

BFA: Brefeldin A Decay
BIMAS: Bioinformatics and Molecular Analysis Section
DC: Dendritic cells
DMSO: Dimethyl Sulfoxide
FCS: Flow Cytometry Software
FLPSDC*FPSV: [A fluorescent standard peptide]
HLA: Human leukocyte antigen
LN: Lymph nodes
MHC: Major histocompatibility complex
PBS: Phosphate-buffered saline
TAMRA: tetramethylrhodamine
TAP: Transporter associated with Antigen
TCR: T-cell receptor
USD: United States Dollar

## Permission to reproduce published materials

Part of Figure 1 has been previously published in [18], Royal Society Interface. The original author may reuse this asset as long as the original work is cited.
