# Supplementary material for: De-risking clinical trial failure through mechanistic simulation
Source: Immunother Adv. 2022 Aug 23;2(1):ltac017. doi: 10.1093/immadv/ltac017 (PMC9514113; doi:10.1093/immadv/ltac017)
Supplement: ltac017_suppl_Supplementary_Appendices [file ltac017_suppl_supplementary_appendices.docx]

Appendix A: Range of parameter values matching results of IMA901

Results presented in Results: Simulated clinical trial results for IMA901 phases I-III are fits of the model and simulated trial to the data of IMA901. However, the presented patient parameter distribution is not the only possible fit, but rather are the points of maximum density in a posterior distribution obtained from Approximate Bayesian Computation. The method used to generate this distribution is detailed in Appendix B: Approximate Bayesian Computation Approach. Figure S1 shows density plots of 2D projections of the resulting posterior distribution, where darker regions indicate a better fit to the data. Critically, the distribution shows all reasonable patient parameter distribution parameters that *could* fit the data, and this allows for the difference between fits to each phase of the clinical trials to be visualised. This difference can be made clearer by starting a fit from a particular known solution and allowing only two parameters to change a time, so that density plots are free of interference from other parameters, as shown in Figure S2. Results show that the patient parameters for phase I are slightly different from phase II and III. This result is surprising; as described in Discussion, it is Phase III that was expected to be different as every patient was given Sunitinib in that phase. Potential reasons are discussed in the main text.

The framework utilised here was useful given the large number of parameters and that different parameter values can lead to a similar fit. We instead obtained the distribution of possible parameter values that can fit the data, checked for consistency of the model with data, and found critical model parameters. Approximate Bayesian Computation was used here, in the absence of a likelihood function. For other models, another Bayesian technique may be more appropriate, but the same approach may be taken.

Appendix B: Approximate Bayesian Computation Approach

Approximate Bayesian Computation may be used to find the range of patient parameters that give virtual clinical trial results consistent with IMA901. The procedure to find these values is presented graphically in Figure 1C and proceeds as follows, where list numbers correspond to the figure:

**1.** Patient parameters (*P*, the lymph transit time, *p* the spread in transit times, *A*, the proportion of dendritic cell MHC-I available to vaccine peptides in the dermis and *T*, the number of cognate antigen-specific T-cells in the lymph node, are shown) are drawn from Gaussian distributions. These distributions are in turn described by their means *µ_i_* and standard deviations *σ_i_*, which are initially unknown. The (prior) distributions for the means and standard deviations are initially flat (uniform), *i.e.* any value with a range is equally likely to be drawn. For each sample Φ*_i_*, a random value of the mean and standard deviation of each parameter distribution is drawn, such that each sample contains a Gaussian distribution for each patient parameter.

2. A virtual cohort of patients (*e.g.* 27 in phase I) is formed from each sample, each taking random parameter values (*e.g.* lymph transit time) from the parameter distributions. A virtual trial is run with each cohort, leading to a prediction of the number of patients that respond to 0, 1, 2 or 3 peptides. The difference between these virtual results and IMA901’s results yields a score *S_i_*:

$$S_{i}=\sum_{i=0}^{5+} \left(y_{i}-\widetilde{y}_{i}\right)^{2}\text{,}$$

where *y_i_* is the number of patients predicted to respond to *i* peptides (or precisely, between *i* − 0.5 and *i* + 0.5 peptides) in the virtual trial and $\widetilde{y}_{i}$ is the number in IMA901. An initial target score of $\frac{\sum_{i} \widetilde{y}_{i}^{2}}{2}$ (=364 for phase I) is selected. Scores below the threshold are accepted. Those above are rejected.

3. The accepted samples Φ*_i_* form the new probability distributions used to select parameter means and standard deviations. In other words, the next sample of means and standard deviations will be closer to the ‘good’ values in the previous sample. Then, the 75th percentile of the sample scores *S_i_* is used to define a new target score.

4. This process is repeated 25 times with 10,000 (accepted) samples per iteration, iteratively updating the posterior distribution. More precisely, we are hence utilising ABC-*Population Monte Carlo* to find a final posterior distribution of patient parameter values that give virtual clinical trial results consistent with IMA901.

Appendix C: Simulated clinical trial results using pMHC-I off-rates estimated from BIMAS and NetMHC 4.0

Simulated results presented in Figure 3 and Figure 5 use measured values of the off-rates of the IMA901 peptides from MHC-I. Had estimated values obtained from utilities such as BIMAS or NetMHC 4.0 been used, the results shown in Figure S3 would be obtained. In this case, it was not possible to match the results of IMA901. The best fit for BIMAS had too few virtual patients responding to three peptides. Attempting to manually change the mean proportion of MHC-I bound to peptide in the dermis to increase the number of responses, however, reduced the number of patients responding to 0 or 1 peptides without increasing the number who respond to 3, making a good fit impossible. This is caused by the difference between the top two and the third best off-rates being too large, in tandem with sharp transitions in the probability of T-cell activation as a function of off-rate (see Figure 2D). The results using estimates from NetMHC 4.0 yield similar problems.
